# Interactive effects of dietary grain and supplemental fat sources on growth performance and carcass characteristics of broiler chickens

**DOI:** 10.1016/j.psj.2025.105076

**Published:** 2025-03-19

**Authors:** Mengzhu Wang, Shemil MacElline, Peter H. Selle, Sonia Y. Liu, Mehdi Toghyani

**Affiliations:** a School of Life and Environmental Sciences, Faculty of Science, The University of Sydney, Camperdown, NSW 2006, Australia; b Poultry Research Foundation, The University of Sydney, Camden, NSW 2570, Australia; c Sydney School of Veterinary Science, The University of Sydney, Camperdown, NSW 2006, Australia

**Keywords:** Metabolizable energy, Supplemental fat, Feed grain, Nutrient digestibility

## Abstract

The current study investigated if grain type or supplemental fat sources affect or interact for growth performance and nutrients digestibility in broiler chickens offered starter and grower diets with lower metabolizable energy (ME) than breeder recommendations. The experiment was conducted using a completely randomized design with a 3 × 3 factorial arrangement of treatments with three grains (wheat, sorghum, barley) and three fat sources (canola oil, poultry fat, and beef tallow), resulting in nine treatments, each replicated eight times with 25 birds per replicate. Diets were fed in starter (0-10 days), grower (10-22 days), finisher (22-35 days), and withdrawal (35-42 days) phases. Starter and grower diets were formulated to 2875 and 2975 kcal/kg ME, 100 and 75 kcal/kg lower than breeder recommendations. Nutrient digestibility was assessed on day 33, and carcass characteristics were evaluated on day 42 of the trial. There was no significant effect of grain or fat source on body weight gain and feed conversion ratio (FCR) over the starter and grower phases (*P* > 0.05). There were no interactions of feed grain and fat sources on overall growth performance and age to 2.5 kg body weight (*P* > 0.05). As the main effect, sorghum-based diets significantly increased overall FCR (0-42 days) compared to wheat- and barley-based diets (*P* < 0.05). Regardless of fat source, barley-based diets decreased breast meat yield and increased fat pad deposition (*P* < 0.05). Sorghum-based diets resulted in the lowest ileal starch digestibility (*P* < 0.05). An interaction between grain and fat source (*P* < 0.01) showed that poultry fat and beef tallow in wheat-based diets improved crude protein digestibility compared to sorghum- and barley-based diets. In summary, these results indicate that all three grain and fat sources tested in this study can be incorporated into broiler chicken diets without significantly impacting growth rate. However, sorghum-based diets, irrespective of the supplemental fat source, result in lower starch digestibility and approximately a 2-point increase in feed conversion.

## Introduction

In modern broiler production, feed accounts for approximately 60-70 % of total production costs, with metabolizable energy (ME) being the most expensive feed component (Noblet et al., 2022). Dietary ME is primarily sourced from starch provided by grains and supplemental fats in the form of added dietary fat. While maize/corn and wheat are the most commonly used grains in broiler diets, alternative grains like barley and sorghum are utilized in Australia due to their availability and comparable nutritional value (Abdollahi et al., 2021; Cowieson and Ravindran, 2008). Barley (*Hordeum vulgare* L.), the fourth most widely produced grain worldwide, can serve as an energy source in poultry diets (Toghyani et al., 2022). While it is commonly used in swine and layer hen diets, its inclusion in broiler diets is limited due to its high fiber content, lower ME, and high levels of soluble non-starch polysaccharides (NSP), particularly beta-glucans (Jacob and Pescatore, 2012). On a dry matter basis, barley contains 33.3 % and 55.2 % more crude fiber and 46.5 % and 95.0 % higher soluble NSP compared to wheat and maize/corn, respectively (Choct, 2006; Knudsen, 2014). Sorghum is a major grain crop in Australia, predominantly grown in Queensland and New South Wales, with an average annual production of 2.1–2.5 million metric tons. The Australian chicken meat industry is one of the largest consumers of grain sorghum, utilizing it as a key energy source in broiler diets due to its high starch content (Selle et al., 2021). However, energy utilization in broiler chickens fed sorghum-based diets is relatively lower due to incomplete starch digestion. This reduced digestibility is influenced by three key ‘starch-extrinsic’ factors in sorghum: kafirin (the dominant protein fraction), non-tannin phenolic compounds, and phytate. These components interfere with starch accessibility and enzyme activity, ultimately compromising starch digestibility and energy utilization in broiler chickens (Liu et al., 2015b; Selle et al., 2021).

Fats and oils, the most energy-dense feed ingredients, supplement grain-based diets to meet ME requirements, offering energy values approximately 2.5 times higher than carbohydrates or proteins (NRC 1994). While canola oil is the primary fat source used in poultry diets in Australia, poultry fat and beef tallow are also used when economically feasible. However, the ME values and digestibility of these fats vary. Canola oil, being rich in unsaturated fatty acids, is more readily digested and absorbed by broilers, leading to higher ME values, while beef tallow and poultry fat, which contain higher levels of saturated fatty acids, exhibit lower digestibility and consequently reduced ME values (Aardsma et al., 2017). For instance, Meng et al. (2004) reported that diets containing tallow had significantly lower apparent fat digestibility and dietary ME content compared to those with canola oil. The degree of saturation in these fats sources influences their utilization, where unsaturated fats, like those in canola oil, are more efficiently utilized by broilers, especially in the early stages of life, compared to saturated fats found in beef tallow and poultry fat (Ravindran et al., 2016).

To optimize performance in line with advancements in genetics, nutrition, and management, major meat chicken breeders continuously update their recommendations for dietary specifications. For instance, Aviagen reduced ME requirements for Ross 308 broilers by 25 and 50 kcal/kg during the starter and grower stages, respectively, in their 2022 recommendations compared to 2019 (Aviagen 2022, 2019). Despite these reductions, literature suggests further potential for decreasing dietary ME without compromising key performance metrics such as growth rate and feed efficiency. Strifler et al. (2023) demonstrated successful reductions of nearly 100 kcal/kg in low crude protein diets for broilers, maintaining FCR over a 41-day growth period. Similarly, in our previous feeding studies, it was confirmed that reducing dietary ME by 100 and 75 kcal/kg in starter and grower phases does not compromise body weight gain, growth rate, feed efficiency (Toghyani et al., 2024; Xie et al., 2024) and nutrient digestibility (Toghyani et al., 2025). However, in those studies all the diets were wheat-based with canola oil. Thus, the current study was designed to investigate if the grain or supplemental fat sources affect or interact for growth parameters when same level of ME reduction is applied to starter and grower diets for male broiler chickens in a 42-day feeding trial.

## Materials and methods

### Birds and experimental design

The experimental protocols and procedures for the present study were approved by the University of Sydney Animal Ethic Committee (protocol number AEC2022/2185). The experiment was conducted using a completely randomized design with a 3 × 3 factorial arrangement of treatments. The experimental factors included three sources of grains (wheat, sorghum and barley) and three supplemental fat sources (canola oil; **CO**, poultry fat; **PF** and beef tallow; **BT**) creating 9 dietary treatments. Each diet was fed to 8 replicate floor pens of 25 Ross 308 off-sex male birds for starter (0-10 d), grower (10 to 22 d), finisher (22 to 35 d) and withdrawal (35 to 42 d) phases. The composition, calculated and analyzed (crude protein, crude fat and starch) nutrient specifications of the experimental diets are listed in Table 1, Table 2, Table 3, Table 4.

**Table 1:** Composition and specification of the starter diets (0-10 d).

Ingredients (%)	Wheat			Sorghum			Barley		
	CO	PF	BT	CO	PF	BT	CO	PF	BT
Wheat 12 %	66.7	66.23	66.06	10.00	10.00	10.00	49.50	49.37	48.91
Sorghum 10 %	-	-	-	50.27	50.49	50.37	-	-	-
Barley 9 %	-	-	-	-	-	-	15.00	15.00	15.00
Canola Oil	0.515	-	-	0.500	-	-	1.551	-	-
Poultry Fat	-	0.547	-	-	0.505	-	-	1.649	-
Beef Tallow	-	-	0.659	-	-	0.607	-	-	1.980
Soybean Meal 46.0 %	22.06	22.07	22.13	28.44	28.18	28.21	22.80	22.84	22.97
Canola Meal 37.5 %	5.00	5.00	5.00	5.00	5.00	5.00	5.00	5.00	5.00
Meat Meal 47 %	3.30	3.30	3.30	3.50	3.50	3.50	3.30	3.30	3.30
Limestone 38 %	0.603	0.603	0.603	0.530	0.532	0.532	0.596	0.596	0.595
Lysine-HCl	0.539	0.539	0.538	0.397	0.406	0.405	0.520	0.519	0.516
DL-Methionine	0.360	0.360	0.360	0.348	0.350	0.350	0.376	0.376	0.377
Na Bicarbonate	0.338	0.338	0.337	0.312	0.316	0.316	0.310	0.310	0.308
L-Threonine	0.218	0.218	0.217	0.151	0.154	0.154	0.218	0.218	0.218
Vit/Min premix^1^	0.200	0.200	0.200	0.200	0.200	0.200	0.200	0.200	0.200
L-Arginine	0.164	0.163	0.163	0.061	0.069	0.068	0.168	0.167	0.165
Salt	0.114	0.114	0.115	0.140	0.138	0.138	0.119	0.119	0.120
Choline Chloride	0.100	0.100	0.100	0.100	0.100	0.100	0.100	0.100	0.100
L-Valine	0.098	0.098	0.098	0.016	0.020	0.020	0.109	0.109	0.109
L-Isoleucine	0.080	0.080	0.079	-	-	-	0.093	0.093	0.092
Xylanase	0.020	0.020	0.020	0.020	0.020	0.020	0.020	0.020	0.020
Phytase	0.020	0.020	0.020	0.020	0.020	0.020	0.020	0.020	0.020
*Calculated and analyzed (protein, fat, starch) specifications*									
AME Kcal/kg	2875	2875	2875	2875	2875	2875	2875	2875	2875
NE^2^ Kcal/kg	2249	2250	2250	2248	2248	2249	2259	2259	2262
Crude Protein %	22.7	22.5	22.8	23.3	23.3	23.5	22.5	22.2	21.9
Dig.Lys. %	1.320	1.320	1.320	1.320	1.320	1.320	1.320	1.320	1.320
Dig.Met.%	0.643	0.643	0.643	0.664	0.665	0.665	0.654	0.654	0.654
Dig.*M* + *C*.%	1.003	1.003	1.003	1.003	1.003	1.003	1.003	1.003	1.003
Dig.Thr.%	0.884	0.884	0.884	0.884	0.884	0.884	0.884	0.884	0.884
Dig.Ile.%	0.884	0.884	0.884	0.888	0.884	0.884	0.884	0.884	0.884
Dig.Leu.%	1.397	1.397	1.398	1.660	1.654	1.654	1.373	1.373	1.374
Dig.Trp.%	0.240	0.240	0.240	0.247	0.246	0.246	0.235	0.235	0.236
Dig.Arg.%	1.399	1.399	1.399	1.399	1.399	1.399	1.399	1.399	1.399
Dig.Val.%	1.003	1.003	1.003	1.003	1.003	1.003	1.003	1.003	1.003
Crude fat %	2.29	2.43	2.51	2.87	2.94	2.92	3.28	3.46	3.63
Starch %	37.5	38.3	37.8	35.0	35.9	34.8	36.1	36.8	36.2
Ca %	0.950	0.950	0.950	0.950	0.950	0.950	0.950	0.950	0.950
Available P %	0.500	0.500	0.500	0.501	0.500	0.500	0.501	0.501	0.501
DEB^3^ meq/kg	210	210	210	230	229	229	213	213	213
PDI^4^ %	90.1	93.7	90.1	75.2	79.0	58.2	79.2	84.7	69.4

CO: canola oil, PF: poultry fat, BT: beef tallow.

1 Vitamin concentrate supplied per kilogram of diet: retinol, 12000 IU; cholecalciferol, 5000 IU; tocopheryl acetate, 75 mg, menadione, 3 mg; thiamine, 3 mg; riboflavin, 8 mg; niacin, 55 mg; pantothenate, 13 mg; pyridoxine, 5 mg; folate, 2 mg; cyanocobalamin, 16 μg; biotin, 200 μg; cereal-based carrier, 149 mg; mineral oil, 2.5 mg. Trace mineral concentrate supplied per kilogram of diet: Cu (sulphate), 16 mg; Fe (sulphate), 40 mg; I (iodide), 1.25 mg; Se (selenate), 0.3 mg; Mn (sulphate and oxide), 120 mg; Zn (sulphate and oxide), 100 mg; cereal-based carrier, 128 mg; mineral oil, 3.75 mg

2 NE calculated as: NE = 0.808 × AMEn (MJ/kg) – 0.017 × Crude Protein (%) + 0.031 × EE (%) (Wu et al., 2019).

3 Dietary electrolyte balance.

4 Pellet durability index determined in triplicate.

**Table 2:** Composition and specification of the grower diets (11-22 d).

Ingredients (%)	Wheat			Sorghum			Barley		
	CO	PF	BT	CO	PF	BT	CO	PF	BT
Wheat 12 %	67.00	66.87	66.55	10.00	10.00	10.00	40.03	39.83	39.16
Sorghum 10 %	-	-	-	53.03	53.09	53.23	-	-	-
Barley 9 %	-	-	-	-	-	-	25.00	25.00	25.00
Canola Oil	0.500	-	-	0.500	-	-	2.150	-	-
Poultry Fat	-	0.539	-	-	0.500	-	-	2.286	-
Beef Tallow	-	-	0.664	-	-	0.500	-	-	2.750
Soybean Meal 46.0 %	19.17	19.28	19.50	22.60	22.59	22.56	19.38	19.45	19.67
Canola Meal 37.5 %	5.00	5.00	5.00	5.00	5.00	5.00	5.00	5.00	5.00
Canola Seeds	4.00	4.00	4.00	4.00	4.00	4.00	4.00	4.00	4.00
Meat Meal 47 %	1.65	1.65	1.65	2.00	2.00	2.00	1.65	1.65	1.65
Limestone 38 %	0.813	0.812	0.811	0.717	0.717	0.717	0.808	0.808	0.807
Lysine-HCl	0.430	0.427	0.420	0.382	0.383	0.383	0.429	0.427	0.422
DL-Methionine	0.286	0.285	0.284	0.301	0.301	0.300	0.321	0.321	0.321
Na Bicarbonate	0.327	0.325	0.322	0.344	0.344	0.345	0.295	0.294	0.292
L-Threonine	0.151	0.149	0.147	0.123	0.123	0.123	0.165	0.165	0.163
Vit/Min Premix^1^	0.200	0.200	0.200	0.200	0.200	0.200	0.200	0.200	0.200
L-Arginine	0.122	0.119	0.114	0.103	0.103	0.103	0.156	0.155	0.151
Salt	0.140	0.141	0.143	0.135	0.135	0.135	0.137	0.138	0.140
Choline Chloride	0.090	0.090	0.090	0.090	0.090	0.090	0.090	0.090	0.090
L-Valine	0.041	0.040	0.037	0.007	0.007	0.007	0.076	0.076	0.075
L-Isoleucine	0.036	0.034	0.031	-	-	-	0.073	0.073	0.071
Xylanase	0.020	0.020	0.020	0.020	0.020	0.020	0.020	0.020	0.020
Phytase	0.020	0.020	0.020	0.020	0.020	0.020	0.020	0.020	0.020
*Calculated and analyzed (protein, fat, starch) specifications*									
AME Kcal/kg	2975	2975	2975	2975	2975	2975	2975	2975	2975
NE^2^ Kcal/kg	2346	2346	2347	2350	2350	2350	2362	2363	2366
Crude Protein %	21.3	21.1	21.5	20.8	21.1	21.3	20.5	20.2	20.6
Dig.Lys. %	1.180	1.180	1.180	1.180	1.180	1.180	1.180	1.180	1.180
Dig.Met.%	0.562	0.562	0.562	0.598	0.598	0.597	0.584	0.584	0.584
Dig.*M* + *C*.%	0.920	0.920	0.920	0.920	0.920	0.920	0.920	0.920	0.920
Dig.Thr.%	0.791	0.791	0.791	0.791	0.791	0.791	0.791	0.791	0.791
Dig.Ile.%	0.802	0.802	0.802	0.802	0.802	0.802	0.802	0.802	0.802
Dig.Leu.%	1.333	1.335	1.340	1.526	1.526	1.527	1.267	1.268	1.270
Dig.Trp.%	0.232	0.232	0.233	0.223	0.223	0.223	0.219	0.219	0.219
Dig.Arg.%	1.274	1.274	1.274	1.274	1.274	1.274	1.274	1.274	1.274
Dig.Val.%	0.909	0.909	0.909	0.909	0.909	0.909	0.909	0.909	0.909
Crude fat %	3.7	3.9	4.1	4.4	4.5	4.3	5.3	5.5	5.8
Starch %	38.2	38.7	38.1	36.6	37.5	36.4	35.8	35.4	35.7
Ca %	0.850	0.850	0.850	0.850	0.850	0.850	0.850	0.850	0.850
Available P %	0.426	0.426	0.426	0.425	0.425	0.425	0.425	0.425	0.425
DEB^3^ meq/kg	208	208	209	216	216	216	210	210	210
PDI^4^ %	83.8	85.6	74.9	34.1	58.2	26.6	63.5	75.8	60.5

CO: canola oil, PF: poultry fat, BT: beef tallow.

1 Vitamin concentrate supplied per kilogram of diet: retinol, 12000 IU; cholecalciferol, 5000 IU; tocopheryl acetate, 75 mg, menadione, 3 mg; thiamine, 3 mg; riboflavin, 8 mg; niacin, 55 mg; pantothenate, 13 mg; pyridoxine, 5 mg; folate, 2 mg; cyanocobalamin, 16 μg; biotin, 200 μg; cereal-based carrier, 149 mg; mineral oil, 2.5 mg. Trace mineral concentrate supplied per kilogram of diet: Cu (sulphate), 16 mg; Fe (sulphate), 40 mg; I (iodide), 1.25 mg; Se (selenate), 0.3 mg; Mn (sulphate and oxide), 120 mg; Zn (sulphate and oxide), 100 mg; cereal-based carrier, 128 mg; mineral oil, 3.75 mg.

2 NE calculated as: NE = 0.808 × AMEn (MJ/kg) – 0.017 × Crude Protein (%) + 0.031 × EE (%) (Wu et al., 2019).

3 Dietary electrolyte balance.

4 Pellet durability index determined in triplicate.

**Table 3:** Composition and specification of the finisher diets (23-35 d).

Ingredients (%)	Wheat			Sorghum			Barley		
	CO	PF	BT	CO	PF	BT	CO	PF	BT
Wheat 12 %	64.52	64.17	63.48	10.00	10.00	10.00	32.81	32.36	31.06
Sorghum 10 %	-	-	-	53.52	53.22	52.68	-	-	-
Barley 9 %	-	-	-	-	-	-	30.00	30.00	30.00
Canola Oil	2.95	-	-	2.50	-	-	4.90	-	-
Poultry Fat	-	3.25	-	-	2.75	-	-	5.25	-
Beef Tallow	-	-	3.75	-	-	3.20	-	-	6.20
Soybean Meal 46.0 %	15.15	15.20	15.40	16.15	16.20	16.30	14.70	14.80	15.15
Canola Meal 37.5 %	6.00	6.00	6.00	6.00	6.00	6.00	6.00	6.00	6.00
Canola Seeds	6.00	6.00	6.00	6.00	6.00	6.00	6.00	6.00	6.00
Celite	2.00	2.00	2.00	2.00	2.00	2.00	2.00	2.00	2.00
Meat Meal 47 %	0.750	0.750	0.750	1.20	1.20	1.20	0.750	0.750	0.750
Limestone 38 %	0.823	0.823	0.822	0.714	0.714	0.713	0.821	0.820	0.820
Lysine-HCl	0.415	0.415	0.415	0.440	0.440	0.435	0.435	0.435	0.430
DL-Methionine	0.265	0.265	0.265	0.295	0.295	0.295	0.310	0.310	0.315
Na Bicarbonate	0.285	0.285	0.280	0.335	0.330	0.330	0.255	0.255	0.250
L-Threonine	0.135	0.135	0.135	0.135	0.135	0.135	0.160	0.160	0.160
Vit/Min Premix^1^	0.200	0.200	0.200	0.200	0.200	0.200	0.200	0.200	0.200
L-Arginine	0.144	0.144	0.143	0.185	0.185	0.185	0.204	0.203	0.199
Salt	0.155	0.155	0.155	0.130	0.130	0.130	0.145	0.145	0.150
Choline Chloride	0.070	0.070	0.070	0.070	0.070	0.070	0.070	0.070	0.070
L-Valine	0.046	0.046	0.046	0.044	0.044	0.045	0.098	0.098	0.098
L-Isoleucine	0.048	0.048	0.048	0.046	0.046	0.046	0.104	0.104	0.103
Xylanase	0.020	0.020	0.020	0.020	0.020	0.020	0.020	0.020	0.020
Phytase	0.020	0.020	0.020	0.020	0.020	0.020	0.020	0.020	0.020
*Calculated and analyzed (protein, fat, starch) specifications*									
AME Kcal/kg	3100	3100	3100	3100	3100	3100	3100	3100	3100
NE^2^ Kcal/kg	2478	2479	2483	2481	2482	2486	2498	2500	2507
Crude Protein %	19.5	19.1	19.6	18.5	18.7	18.9	18.3	17.8	17.8
Dig.Lys. %	1.080	1.080	1.080	1.080	1.080	1.080	1.080	1.080	1.080
Dig.Met.%	0.523	0.523	0.524	0.565	0.565	0.566	0.551	0.552	0.553
Dig.*M* + *C*.%	0.864	0.864	0.864	0.864	0.864	0.864	0.864	0.864	0.864
Dig.Thr.%	0.724	0.724	0.724	0.724	0.724	0.724	0.724	0.724	0.724
Dig.Ile.%	0.745	0.745	0.745	0.745	0.745	0.745	0.745	0.745	0.745
Dig.Leu.%	1.216	1.216	1.215	1.358	1.357	1.355	1.121	1.120	1.121
Dig.Trp.%	0.213	0.213	0.213	0.194	0.194	0.194	0.194	0.194	0.194
Dig.Arg.%	1.177	1.177	1.177	1.177	1.177	1.177	1.177	1.177	1.177
Dig. Val.%	0.842	0.842	0.842	0.842	0.842	0.842	0.842	0.842	0.842
Crude fat %	6.6	7.1	7.6	7.1	7.4	7.6	8.5	9.0	9.6
Starch %	36.6	37.2	36.4	36.9	37.6	36.1	34.2	34.7	33.6
Ca %	0.750	0.750	0.750	0.750	0.750	0.750	0.750	0.750	0.750
Available P %	0.378	0.378	0.377	0.377	0.377	0.377	0.376	0.375	0.375
DEB^3^ meq/kg	189	189	189	189	189	189	189	189	189
PDI^4^ %	77.4	82.6	79.3	46.0	44.5	41.3	77.7	84.0	79.2

C CO: canola oil, PF: poultry fat, BT: beef tallow.

1 Vitamin concentrate supplied per kilogram of diet: retinol, 12000 IU; cholecalciferol, 5000 IU; tocopheryl acetate, 75 mg, menadione, 3 mg; thiamine, 3 mg; riboflavin, 8 mg; niacin, 55 mg; pantothenate, 13 mg; pyridoxine, 5 mg; folate, 2 mg; cyanocobalamin, 16 μg; biotin, 200 μg; cereal-based carrier, 149 mg; mineral oil, 2.5 mg. Trace mineral concentrate supplied per kilogram of diet: Cu (sulphate), 16 mg; Fe (sulphate), 40 mg; I (iodide), 1.25 mg; Se (selenate), 0.3 mg; Mn (sulphate and oxide), 120 mg; Zn (sulphate and oxide), 100 mg; cereal-based carrier, 128 mg; mineral oil, 3.75 mg;

2 NE calculated as: NE = 0.808 × AMEn (MJ/kg) – 0.017 × Crude Protein (%) + 0.031 × EE (%) (Wu et al., 2019);

3 Dietary electrolyte balance;

4 Pellet durability index determined in triplicate.

**Table 4:** Composition and specification of the withdrawal diets (36-42 d).

Ingredients (%)	Wheat			Sorghum			Barley		
	CO	PF	BT	CO	PF	BT	CO	PF	BT
Wheat 12 %	69.12	68.87	68.41	10.00	10.00	10.00	32.14	31.80	30.65
Sorghum 10 %	-	-	-	58.14	57.89	57.65	-	-	-
Barley 9 %	-	-	-	-	-	-	35.0	35.0	35.0
Canola Oil	2.05	-	-	1.55	-	-	4.30	-	-
Poultry Fat	-	2.25	-	-	1.75	-	-	4.50	-
Beef Tallow	-	-	2.60	-	-	1.95	-	-	5.45
SBM 46.0 %	11.90	11.95	12.05	13.00	13.05	13.10	11.40	11.45	11.70
Canola Meal 37.5 %	7.00	7.00	7.00	7.00	7.00	7.00	7.00	7.00	7.00
Canola Seeds	7.00	7.00	7.00	7.00	7.00	7.00	7.00	7.00	7.00
Meat Meal 47 %	0.500	0.500	0.500	0.800	0.800	0.800	0.500	0.500	0.500
Limestone 38 %	0.764	0.764	0.764	0.700	0.700	0.700	0.762	0.762	0.761
Lysine-HCl	0.410	0.410	0.410	0.440	0.440	0.435	0.435	0.435	0.430
DL-Methionine	0.220	0.220	0.220	0.255	0.255	0.255	0.275	0.275	0.280
Na Bicarbonate	0.265	0.265	0.265	0.325	0.325	0.320	0.230	0.230	0.230
L-Threonine	0.120	0.120	0.120	0.120	0.120	0.120	0.150	0.150	0.150
Vit/Min Premix^1^	0.200	0.200	0.200	0.200	0.200	0.200	0.200	0.200	0.200
L-Arginine	0.151	0.150	0.149	0.199	0.198	0.197	0.220	0.219	0.217
Salt	0.140	0.140	0.145	0.110	0.110	0.115	0.130	0.130	0.135
Choline Chloride	0.060	0.060	0.060	0.060	0.060	0.060	0.060	0.060	0.060
L-Valine	0.025	0.026	0.026	0.025	0.025	0.025	0.086	0.087	0.087
L-Isoleucine	0.038	0.038	0.038	0.037	0.037	0.037	0.103	0.103	0.103
Xylanase	0.020	0.020	0.020	0.020	0.020	0.020	0.020	0.020	0.020
Phytase	0.020	0.020	0.020	0.020	0.020	0.020	0.020	0.020	0.020
*Calculated and analyzed (protein, fat, starch) specifications*									
AME Kcal/kg	3150	3150	3150	3150	3150	3150	3150	3150	3150
NE^2^ Kcal/kg	2517	2518	2521	2520	2521	2523	2540	2542	2549
Crude Protein %	19.2	18.9	19.1	17.9	18.1	18.3	17.6	17.4	17.6
Dig.Lys. %	1.027	1.027	1.027	1.027	1.027	1.027	1.027	1.027	1.027
Dig.Met.%	0.478	0.478	0.479	0.523	0.524	0.524	0.511	0.511	0.513
Dig.*M* + *C*.%	0.822	0.822	0.822	0.822	0.822	0.822	0.822	0.822	0.822
Dig.Thr.%	0.688	0.688	0.688	0.688	0.688	0.688	0.688	0.688	0.688
Dig.Ile.%	0.709	0.709	0.709	0.709	0.709	0.709	0.709	0.709	0.709
Dig.Leu.%	1.177	1.177	1.176	1.329	1.328	1.327	1.065	1.065	1.064
Dig.Trp.%	0.206	0.206	0.206	0.185	0.185	0.185	0.184	0.184	0.184
Dig.Arg.%	1.130	1.130	1.130	1.130	1.130	1.130	1.130	1.130	1.130
Dig.Val.%	0.801	0.801	0.801	0.801	0.801	0.801	0.801	0.801	0.801
Crude fat %	6.2	6.6	6.9	6.7	6.9	6.9	8.4	8.8	9.3
Starch %	39.6	39.9	39.2	40.5	41.2	39.8	36.3	36.9	35.9
Ca %	0.700	0.700	0.700	0.700	0.700	0.700	0.700	0.700	0.700
Available P %	0.367	0.367	0.367	0.358	0.358	0.358	0.365	0.364	0.364
DEB^3^meq/kg	182	182	182	182	182	182	182	182	182
PDI^4^ %	80.8	83.5	82.0	29.2	23.1	22.7	69.0	71.3	68.2

C CO: canola oil, PF: poultry fat, BT: beef tallow.

1 Vitamin concentrate supplied per kilogram of diet: retinol, 12000 IU; cholecalciferol, 5000 IU; tocopheryl acetate, 75 mg, menadione, 3 mg; thiamine, 3 mg; riboflavin, 8 mg; niacin, 55 mg; pantothenate, 13 mg; pyridoxine, 5 mg; folate, 2 mg; cyanocobalamin, 16 μg; biotin, 200 μg; cereal-based carrier, 149 mg; mineral oil, 2.5 mg. Trace mineral concentrate supplied per kilogram of diet: Cu (sulphate), 16 mg; Fe (sulphate), 40 mg; I (iodide), 1.25 mg; Se (selenate), 0.3 mg; Mn (sulphate and oxide), 120 mg; Zn (sulphate and oxide), 100 mg; cereal-based carrier, 128 mg; mineral oil, 3.75 mg;

2 NE calculated as: NE = 0.808 × AMEn (MJ/kg) – 0.017 × Crude Protein (%) + 0.031 × EE (%) (Wu et al., 2019);

3 Dietary electrolyte balance;

4 Pellet durability index determined in triplicate.

### Diet preparation

Prior to diet formulation, representative subsamples of wheat, barley, and sorghum were analysed by near-infrared spectroscopy to predict proximate analysis, digestible amino acid concentrations, and ME using AMINONIR®PROX, AMINONIR®NIR, and AMINONIR® NRG (Evonik Nutrition & Care, Rodenbacher Chaussee 4, 63457 Hanau-Wolfgang, Germany), respectively. Diets were based on wheat (12.5 % CP; ME 3180 kcal/kg), sorghum (10.0 % CP; ME 3250 kcal/kg), barley (9.0 % CP; ME 2850 kcal/kg) soybean meal (46.0 % CP; ME 2400 kcal/kg), meat and bone meal (47.0 % CP; ME 2000 kcal/kg), solvent canola meal (37.5 % CP; ME 1980 kcal/kg) and canola seed (21.0 % CP; ME 4500 kcal/kg), canola oil (ME 8500 kcal/kg), poultry fat (ME 8200 kcal/kg) and beef tallow (ME 7400 kcal/kg) without any inorganic phosphate sources. To maintain pellet quality and reflect commercial practices in Australia, a minimum of 10 % wheat was included in sorghum-based diets, where sorghum served as the primary grain. Barley was incorporated incrementally based on our prior study (Toghyani et al., 2022), with inclusion levels of 15, 25, 30, and 35 % in the starter, grower, finisher, and withdrawal diets, respectively. The experimental diets were formulated to be isocaloric with same level of digestible lysine applying ideal amino acid/protein ratio recommended by breeder (Aviagen, 2022) without any minimum or maximum set for crude protein. Starter and grower diets were formulated to 2875 and 2975 kcal/kg ME, 100 and 75 kcal/kg lower than breeder recommendations. The finisher and withdrawal diets were formulated to 3100 and 3150 kcal/kg ME, respectively.

Poultry fat and beef tallow were heated to ∼35-40°C to maintain a full liquid status prior to incorporation into the diets, allowing for uniform mixing without compromising fat quality. Wheat, sorghum and barley were mediumly ground (4.0 mm hammer-mill screen). All the diets were steam-pelleted through a Palmer PP330 pellet press (Palmer Milling Engineering, 20-24 Altin street, Griffith, NSW, Australia) at a conditioning temperature of 80°C with a conditioner residence time of 15 s and were then cooled in a vertical cooler to room temperature. All experimental diets included both xylanase (Ronozyme® WX 2000 at 200 g/ton) and phytase (Ronozyme® HiPhorius 10 at 200 g/ton; 2000 FYT/kg of feed) enzymes to align with commercial practices. These diets did not contain any antibiotic growth promoters. Acid in soluble ash (Celite World Minerals, 2500 San Miguelito Rd, Lompoc, CA) was included at 2.0 % in all the finisher diets as an inert marker to determine the digestibility coefficients of starch, protein (N), and fat.

Pellet durability index (PDI) of all diets were determined in triplicate, using the NHP 200 New Holman Automatic Pellet Tester (TekPro Ltd, North Walsham, Norfolk, UK) and results are included in Table 1, Table 2, Table 3, Table 4.

### Birds’ management and data collection

Birds had unrestricted access to feed and water in an environmentally controlled facility throughout the experiment. The lighting program followed breeder recommendations. An initial room temperature of 33 ± 1°C was maintained for the first week, which was gradually decreased to 22 ± 1°C by the end of the third week and maintained at this temperature for the duration of the feeding study.

Birds were weighed on a pen basis on days 0, 10, 22, 35 and 42 to determine body weights (**BW**) and calculate body weight gain (**BWG**). Feed intake (**FI**) was measured in similar intervals and used to calculate feed conversion ratio (**FCR**) for each phase. Mortality was recorded daily, and dead bird's BW was used to correct FCR values. On day 33, a total of 3 birds from each pen were individually weighed and euthanised by intravenous injections of sodium pentobarbitone. The abdominal cavity was opened, digesta samples were collected from the distal ileum. Digesta were gently expressed and pooled for each replicate pen, homogenized, freeze-dried, and ground through 0.5 mm screen and then analysed for starch, protein (N) and crud fat content.

On day 39, all birds in each pen underwent visual inspection to assess footpad dermatitis (FD) and hock burns (lesions) HB on both feet. Any signs of FD were scored from 0 to 4 (Stracke et al., 2021), and HB were scored from 0 to 4 following the guidelines by Butterworth et al. (2009). On day 42 of the experiment, a total of 3 birds per pen were randomly selected and euthanized for carcass analysis. Abdominal fat-pad was removed, weighed and recorded against live BW to calculate relative abdominal fat-pad weights. Also, *Pectoralis* major, *Pectoralis* minor, and leg quarters were removed, weighed and recorded against live BW to calculate relative weights of carcass components. The major breast muscle was visually examined and scored for the presence of woody breast and white striping according to Kuttappan et al. (2016).

### Chemical analysis and calculations

Starch concentrations in diets and digesta samples were determined by using total starch assay kits (Megazyme, Bray Business Park, Bray, Co. Wicklow, Ireland) as described in Mahasukhonthachat et al. (2010). Nitrogen contents of diets and digesta were determined using a nitrogen determinator (Leco Corporation, 3000 Lakeview Avenue, St. Joseph, MI) by the Dumas method. Acid insoluble ash (AIA) concentrations was determined by the method described by Siriwan et al. (1993). Fat content of diets an digesta was determined using methods of AOAC (2005).

The apparent digestibility coefficients for starch, protein (N) and fat were calculated from the following equation:

$$Digestibility\;Coefficient\;=\frac{\left(Nutrient\;Diet÷AIA\;diet\right)\;-\left(Nutriten\;Digesta\;÷AIA\;Digesta\right)}{Nutrient\;Diet÷AIA\;diet}$$

### Statistical analysis

Data were checked for normality and then subjected to statistical analysis using 2-way ANOVA of GLM procedure in JMP®13.0.0 (SAS Institute Inc., JMP Software, 100 SAS Campus Drive, Cary, NC) to assess the main effects of grain and supplemental fat sources and their interaction. Each pen was considered an experimental unit and the values presented in the Tables are means with pooled standard error of mean (**SEM**). If a significant effect of treatment was detected, differences between treatments or main effects were separated by least square differences test (**LSD**). Significance was considered at *P* < 0.05 and *P* < 0.1 was indicated and discussed as a trend.

## Results

Mortality rate in this trial was not affected by dietary treatments and remained below 3.0 % for the overall 42-d period. Based on the data presented in Table 5, over the starter and grower periods, there was no dietary interaction of grain and oil source on BW, FI and FCR. Neither grains nor fat sources had any significant impact on growth performance (*P* > 0.05). Similarly, over the 0-22 d period, grain and oil did not interact for any performance parameters.

**Table 5:** Broilers growth performance over the starter (0-10 d) and grower (10-22 d) periods.

Treatments		Body weight g/b			Feed intake g/b			FCR g/g		
Grain	Fat^1^	D 0	D 10	D 22	0-10 d	10-22 d	0-22 d	0-10 d	10-22 d	0-22 d
Wheat	CO	41.4	334	1294	309	1283	1592	1.056	1.352	1.282
Wheat	PF	41.2	330	1269	305	1258	1562	1.056	1.355	1.284
Wheat	BT	41.4	331	1282	307	1271	1579	1.060	1.352	1.283
Sorghum	CO	41.3	331	1289	309	1278	1587	1.067	1.349	1.283
Sorghum	PF	41.4	330	1272	306	1261	1568	1.063	1.354	1.285
Sorghum	BT	41.7	331	1278	307	1267	1574	1.058	1.354	1.284
Barley	CO	41.8	329	1274	304	1263	1568	1.060	1.352	1.283
Barley	PF	41.5	330	1270	305	1259	1563	1.056	1.356	1.284
Barley	BT	41.6	326	1265	304	1254	1558	1.069	1.350	1.285
SEM		0.23	2.9	10.8	2.20	9.82	11.5	0.007	0.002	0.001
*Main effects*										
Grain										
Wheat		41.3	332	1282	307	1270	1577	1.057	1.353	1.283
Sorghum		41.5	331	1280	307	1269	1576	1.063	1.352	1.284
Barley		41.6	328	1270	304	1258	1562	1.062	1.352	1.284
Fat										
CO		41.5	331	1286	307	1275	1582	1.061	1.351	1.282
PF		41.4	330	1270	305	1259	1564	1.058	1.355	1.284
BT		41.6	329	1275	306	1264	1570	1.063	1.352	1.284
Source of variation *P value*										
Grain source		0.395	0.300	0.276	0.172	0.282	0.231	0.604	0.956	0.767
Fat source		0.596	0.726	0.241	0.495	0.142	0.159	0.755	0.228	0.357
Grain × Fat		0.744	0.731	0.806	0.849	0.855	0.834	0.672	0.801	0.996

Each value for each treatment represents the mean of 3 birds per replicate, and 8 replicates per treatment.

^a-c^Means within a column not sharing a superscript differ significantly at the *P* < 0.05 level for the treatment effects and at the P level shown for the main effects.

1 Co: Canola oil; PF: Poultry fat; BT: Beef tallow.

In the finisher period there was no interaction between grain and supplemental fat source for BW (*P* > 0.05; Table 6). However, as the main effect grain source affected BW at day 35, where birds fed wheat-based diet were heavier and gained more weight during the finisher phase compared to birds fed either sorghum or barley-based diets (*P* < 0.05). Similarly, birds fed wheat-based diets tended (*P* = 0.099) to be heavier at day 42. An interaction between grain and supplemental fat source for FI in finisher (22-35 d), withdrawal (35-42 d) and 0-35 d periods resulted in lower FI in sorghum-based diets only with canola oil (*P* < 0.05). However, considering the entire experimental period (0-42 d), FI was neither affected by dietary factors nor their interactions (*P* > 0.05). During the finisher (22-35 d) and 0-35 day periods, birds fed diets with beef tallow recorded a lower FCR than canola oil, only in wheat-based diets, which led to a significant interaction between grain and fat source (*P* < 0.05). As presented in Table 6, in withdrawal period there was no significant interaction of dietary factors on FCR (*P* > 0.05). Birds fed the sorghum-based diets recorded the highest overall FCR (0-42 d), which was significantly higher than the birds fed wheat and barley-based diets (*P* < 0.05; Table 6). The age to reach to 2.5 kg of live BW was not affected by dietary treatments or their interactions (*P* > 0.05).

**Table 6:** Broilers growth performance over the finisher (23-35 d), withdraw (35-42 d) and overall experimental period (0-42 d).

Treatments		BW g/b		FI g/b				FCR g/g				Age to 2.5 kg BW
Grain	Fat^1^	D 35	D 42	22-35 d	35-42 d	0-35 d	0-42 d	22-35 d	35-42 d	0-35 d	0-42 d	Day
Wheat	CO	2766	3543	2185^a^	1425^a^	3592^a^	5018	1.475^a^	1.831	1.319^ab^	1.433	30.0
Wheat	PF	2725	3480	2114^ab^	1399^ab^	3501^ab^	4900	1.441^ab^	1.865	1.305^ab^	1.426	30.6
Wheat	BT	2776	3491	2075^ab^	1353^ab^	3483^ab^	4836	1.380^b^	1.902	1.274^c^	1.402	30.4
Sorghum	CO	2693	3444	2028^b^	1421^b^	3448^b^	4869	1.434^ab^	1.896	1.301^abc^	1.431	30.9
Sorghum	PF	2706	3435	2126^ab^	1401^ab^	3533^ab^	4934	1.473^a^	1.926	1.356^a^	1.454	31.0
Sorghum	BT	2706	3474	2128^ab^	1407^ab^	3539^ab^	4947	1.481^a^	1.836	1.329^a^	1.441	30.6
Barley	CO	2725	3449	2086^ab^	1383^ab^	3481^ab^	4864	1.427^ab^	1.926	1.297^bc^	1.428	30.9
Barley	PF	2701	3427	2090^ab^	1348^ab^	3475^ab^	4823	1.449^ab^	1.861	1.306^ab^	1.424	31.0
Barley	BT	2705	3460	2098^ab^	1391^ab^	3477^ab^	4868	1.447^ab^	1.851	1.306^ab^	1.425	30.7
SEM		17.7	36.6	26.8	33.7	28.6	47.3	0.019	0.038	0.010	0.009	0.326
*Main effects*												
Grain												
Wheat		2756^a^	3505	2125	1392	3525	4918	1.432	1.866	1.299	1.420^b^	30.3
Sorghum		2702^b^	3451	2094	1410	3507	4916	1.463	1.886	1.318	1.442^a^	30.8
Barley		2710^b^	3445	2091	1374	3478	4851	1.441	1.879	1.303	1.425^b^	30.9
Fat												
CO		2728	3479	2100	1410	3507	4916	1.445	1.884	1.306	1.431	30.6
PF		2711	3447	2110	1383	3503	4885	1.454	1.884	1.312	1.434	30.9
BT		2729	3475	2100	1384	3500	4883	1.436	1.863	1.303	1.422	30.6
Source of variation *P value*												
Grain source		0.007	0.099	0.249	0.435	0.125	0.159	0.155	0.801	0.060	0.024	0.093
Fat source		0.386	0.524	0.869	0.541	0.949	0.627	0.661	0.743	0.492	0.331	0.527
Grain × Fat		0.405	0.838	0.003	0.705	0.009	0.081	0.009	0.189	0.011	0.237	0.847

Each value for each treatment represents the mean of 3 birds per replicate, and 8 replicates per treatment.

^a-c^ Means within a column not sharing a superscript differ significantly at the *P* < 0.05 level for the treatment effects and at the P level shown for the main effects.

1 Co: Canola oil; PF: Poultry fat; BT: Beef tallow.

As summarized in Table 7, no significant interaction of dietary factors was observed for any of carcass yield parameters (*P* > 0.05). However, as the main effect, barley-based diets decreased breast meat yield, while poultry fat increased the yield (*P* < 0.01). Birds fed barley-based diets recorded a higher fat pad weight than the wheat and sorghum-based diets (*P* < 0.01). Canola oil decreased fat pad weight compared to beef tallow (*P* < 0.01). Empty gizzard weight was also affected by both grain and fat source. Feeding sorghum or barley-based diets increased gizzard weight (*P* < 0.01). Birds fed canola oil supplemented diets recorded higher gizzard weight than poultry fat or beef tallow (*P* < 0.01; Table 7). While fat source had no effect on foot pad dermatitis (**FD**) scores in birds fed barley-based diets, canola oil inclusion resulted in the highest FD scores for wheat-based diets but the lowest for sorghum-based diets, leading to a significant grain × fat source interaction (*P* < 0.05). Woody breast, white stripping and hock burn scores were not affected by dietary treatments (*P* > 0.05; Table 7).

**Table 7:** Carcass yield, woody breast (WB), white striping (WS) scores at day 42 post-hatch and foot pad scoring at day 39 post-hatch.

Treatments		Carcass yield g/kg live BW						Breast score^3^		Foot pad^4^	
Grain	Fat^1^	*P*. Major 2	*P*. Minor	*P*. Total	Leg Qtr.	Fat pad	Gizzard	WS	WB	FD	HB
Wheat	CO	199	36.9	236	214	11.70	10.77	1.13	1.93	0.376^ab^	0.865
Wheat	PF	200	37.3	238	211	12.21	10.21	1.21	1.96	0.247^abc^	0.810
Wheat	BT	196	35.3	231	216	12.38	10.19	1.06	1.88	0.108^c^	0.949
Sorghum	CO	192	35.7	227	216	11.97	12.17	1.29	2.00	0.114^c^	0.503
Sorghum	PF	198	36.5	235	212	13.04	11.06	0.96	2.08	0.438^a^	0.943
Sorghum	BT	193	36.2	229	213	13.21	11.45	1.04	1.71	0.329^abc^	0.606
Barley	CO	190	34.7	224	217	13.02	11.90	0.88	1.96	0.189^bc^	0.835
Barley	PF	193	35.6	229	215	13.71	10.92	1.00	1.96	0.213^bc^	0.839
Barley	BT	189	34.4	224	217	14.67	10.92	0.96	1.96	0.202^bc^	0.814
SEM		2.30	0.445	2.38	1.85	0.451	0.245	0.135	0.153	0.0788	0.1668
*Main effects*											
Grain											
Wheat		198^a^	36.5^a^	235^a^	214	12.10^b^	10.39^b^	1.14	1.89	0.244	0.875
Sorghum		194^ab^	36.1^a^	230^a^	214	12.74^b^	11.56^a^	1.10	1.93	0.294	0.684
Barley		191^b^	34.9^b^	226^b^	216	13.80^a^	11.25^a^	0.94	1.96	0.201	0.829
Fat											
CO		193^b^	35.8^ab^	229^b^	215	12.23^b^	11.61^a^	1.10	1.93	0.226	0.735
PF		197^a^	36.5^a^	234^a^	212	12.99^ab^	10.73^b^	1.06	2.00	0.299	0.864
BT		193^b^	35.3^b^	228^b^	215	13.42^a^	10.86^b^	1.02	1.85	0.213	0.790
Source of variation *P value*											
Grain source		0.007	<.001	<.001	0.190	<.001	<.001	0.204	0.855	0.364	0.349
Fat source		0.031	0.004	0.008	0.099	0.007	<.001	0.788	0.475	0.359	0.637
Grain × Fat		0.824	0.161	0.717	0.646	0.787	0.752	0.437	0.706	0.021	0.520

Each value for each treatment represents the mean of 3 birds per replicate, and 8 replicates per treatment.

^a-c^Means within a column not sharing a superscript differ significantly at the *P* < 0.05 level for the treatment effects and at the P level shown for the main effects.

1 Co: Canola oil; PF: Poultry fat; BT: Beef tallow.

2 P: Pectoral major and minor muscle;

3 WS: White striping, WB: Woody breast;

4 FD: Foot pad dermatitis, HB: Hock burn.

The impact of dietary treatments on nutrient digestibility is reported in Table 8. There were no significant differences on crude fat digestibility among dietary treatments (*P* > 0.05). However, as the main effect both grain and fat source significantly affected starch digestibility. With beef tallow inclusion, starch digestibility was reduced compared to poultry fat. Wheat- and barley-based diets also exhibited greater starch digestibility than sorghum-based diets (*P* < 0.01). A significant interaction effect (*P* = 0.008) between grain type and fat source was observed for protein digestibility. The inclusion of poultry fat and beef tallow in wheat-based diets increased crude protein digestibility compared to sorghum- and barley-based diets.

**Table 8:** Ileal digestibility (%) of starch, crude fat and crude protein determined on day 33 of the trial.

Treatments		Nutrients		
Grain	Fat^1^	Starch	Fat	Protein
Wheat	CO	97.9	92.6	77.5^c^
Wheat	PF	99.4	94.1	80.9^ab^
Wheat	BT	96.0	93.6	81.4^a^
Sorghum	CO	91.3	93.4	75.6^c^
Sorghum	PF	91.5	94.1	76.5^c^
Sorghum	BT	91.0	93.8	77.9^bc^
Barley	CO	98.9	94.2	78.2^abc^
Barley	PF	98.8	94.0	76.2^c^
Barley	BT	98.9	94.0	77.3^c^
SEM		0.01	0.33	0.87
Main effects				
Grain				
Wheat		97.8^a^	93.4	79.9
Sorghum		91.3^b^	93.7	76.7
Barley		98.9^a^	94.1	77.2
Fat				
CO		96.1^ab^	93.4	77.1
PF		96.6^a^	94.0	77.8
BT		95.3^b^	93.8	78.9
				
Source of variation *P value*				
Grain source		<.001	0.068	<.001
Fat source		0.045	0.063	0.015
Grain × Fat		0.061	0.148	0.004

Each value for each treatment represents the mean of 3 birds per replicate, and 8 replicates per treatment.

^a-b^Means within a column not sharing a superscript differ significantly at the *P* < 0.05 level for the treatment effects and at the P level shown for the main effects.

1 Co: Canola oil; PF: Poultry fat; BT: Beef tallow.

## Discussion

This study aimed to evaluate the impact of dietary grain type and supplemental fat source on growth performance in broiler chickens fed starter and grower diets with ME than the breeder recommendations. The findings indicate that sorghum- or barley-based diets, when supplemented with canola oil, poultry fat or beef tallow, resulted in comparable BW and feed efficiency to wheat-based diets with canola oil up to the end of the grower phase. In fact, birds in this study outperformed the breeder objectives (Aviagen, 2022) and gained higher weight (+13.1 %; 3467 vs 3066 g/bird) and exhibited a better FCR (- 5.7 %; 1.430 vs 1.517) over the entire production period (0 - 42 d).

In the current study, there were no interactions between grain and supplemental fat source for any performance parameters except for FI and FCR over the finisher period, where FCR improved by 6.4 % (1.380 versus 1.475) with beef tallow supplementation compared with canola oil only in wheat-based diets, this was mainly driven by the lower feed intake in response to beef tallow in wheat-based diets as the BWs on day 35 were almost identical. The reduction in FI observed in wheat-based diets supplemented with beef tallow could be partially attributed to the higher degree of fat saturation in beef tallow compared to canola oil and poultry fat, as well as the greater soluble NSP content in wheat, which may have increased intestinal viscosity, thereby altering gut motility and transit time. This combination likely prolonged gastric retention times, leading to earlier satiety signals and a subsequent reduction in FI. Moreover, this could also explain the higher protein digestibility observed in wheat-based diets with beef tallow. The slower digesta passage rate may have allowed for more efficient enzymatic digestion and nutrient absorption. Since fat and starch digestibility remained unaffected in wheat-based diets with different fat sources, it suggests that ME availability was not limiting. Therefore, the combination of lower FI and improved protein digestibility likely contributed to the better FCR observed in these diets.

The unaffected growth performance during the starter and grower phases could be due to the lower total fat intake, as birds in finisher period had the highest percentage of feed intake, 43 % vs 6.25 % in starter, 22.4 % in grower and 28.4 % in withdrawal. In contrast, Moradi et al. (2024) observed interactions between grain source (wheat and corn) and oil source (soybean oil, fish oil, tallow, and palm oil) for BWG and FCR during the first three weeks of age. The authors reported improvements in BWG and FCR in birds fed wheat-based diets supplemented with tallow. Similarly, Tancharoenrat et al. (2015) reported an interaction between cereal type and fat source for BWG, where fat source had no effect on BWG in birds fed sorghum-based diets. However, in birds fed wheat- or corn-based diets, BWG was higher with soybean oil supplementation compared to tallow supplementation. These inconsistencies might be attributed to the ME values assigned to different fat sources and the variation in fat inclusion levels. For instance, in Moradi et al. (2024), fat inclusion was fixed at 3.0 %, while in Tancharoenrat et al. (2015), it was set at 6.0 %. In contrast, in our study, the fat inclusion rate was not fixed and varied among treatments.

The effect of grain source on FCR of the birds fed sorghum-based diets was age dependent. Birds fed sorghum-based diets exhibited a statistically higher FCR, but this difference was significant only when analyzed over the entire trial period (0-42 day). Similar findings were reported in a previous study examining the impact of age on sorghum-based diets, where complete replacement of corn with sorghum did not negatively impact broiler performance during the initial growth phase until day 21 post-hatch (Torres et al., 2013). This could be attributed to the greater feed intake of older birds and the increased ingestion of anti-nutritive factors in sorghum, including kafirin, phenolic compounds, and phytate (Liu et al., 2015b). Kafirin, which constitutes more than 50 % of the protein content in sorghum, impedes energy utilisation by forming linkage structures with starch (Selle et al., 2020). Torres et al. (2013) indicated that phenolic acids reduce aminopeptidase activity and increases small intestinal cell proliferation, resulting in greater epithelial loss and compromised growth performance particularly in older birds. Tannins are widely regarded as key anti-nutritive factors in sorghum. Nyamambi et al. (2007) reported a linear decline in villus height and crypt depth in broiler chickens with increasing tannin levels. However, tannins may not pose a significant concern in Australian sorghum-based diets, as the cultivars grown locally are predominantly zero-tannin sorghum (Khoddami et al., 2015; Truong, 2017).

The nutritional factors influencing fat-pad deposition in broiler chickens are well-documented and include dietary energy levels, supplemental fat sources, protein and amino acid concentrations, and the use of feed additives such as emulsifiers (Fouad and El-Senousey, 2014). In our study, birds fed barley-based diets deposited higher abdominal fat than those fed wheat- and sorghum-based diets. The barley used in this study had a ME value of 2850 kcal/kg which was fairly lower than that of wheat (3180 kcal/kg) and sorghum (3250 kcal/kg). This resulted in in a higher inclusion of supplemental fat in barley-based diets, which overall was 2 to 5 times higher than that in wheat- and sorghum-based diets and led to higher net energy (**NE**) in barley-based diets. Musigwa et al. (2019) compared diets with identical ME levels and observed that an extra 40 kcal/kg in NE resulted in a 2.8-unit increase in fat-pad yield (1.04 % vs. 0.76 %) despite similar ME levels. Additionally, the efficiency of converting ME to NE varies across energy sources, with fats having a lower heat increment than carbohydrates and proteins, leading to higher NE efficiency (Wu et al., 2019). Energy partitioning between protein accretion and fat deposition depends on the balance between energy intake and the bird's maintenance and production requirements (Toghyani et al., 2024). Given this, the higher NE levels in barley-based diets, while maintaining a constant digestible amino acid intake, likely contributed to the increased fat pad weights observed in this study.

The fatty acid profiles and the degree of saturation in different fat sources could also affect fat pad deposition in meat chickens (Crespo and Esteve-Garcia, 2001). In this study, birds fed diets supplemented with beef tallow exhibited higher abdominal fat deposition, while those receiving poultry fat showed an intermediate response, not significantly different from either canola oil or beef tallow. Similarly, other researchers have also reported higher abdominal fat pad with animal fat sources compared to plant sourced oils with a lower degree of saturation (Brue and Latshaw, 1985; Poorghasemi et al., 2013). The saturated fatty acids compositions in total fatty acids have been determined to be 55.15 % in beef tallow, which is 1.7 times higher than poultry fat (32.48 %) and nearly 7 times than canola oil (7.94 %) (Farahmandfar et al., 2015; Naquiah et al., 2013). Compared to saturated fatty acids, unsaturated fatty acids may reduce abdominal fat pad deposition due to reduced hepatic lipogenesis by depressing the fatty acids synthetase activity, and a greater rate of -oxidation by higher specific activity of heart carnitine palmitoyl transferase I and L-3-hydroxyacyl-CoA dehydrogenase (Sanz et al., 2000). Also, the ME values of animal fat is more variable than plant originated oils. For example, the ME value of tallow has been reported to range from 5448 to 8118 kcal/kg, while that of soybean oil reported to be lower, ranging from 6665 to 8796 kcal/kg in broiler chickens (Thng et al., 2020).

Gizzard is the primary muscular organ responsible for mechanical digestion in poultry, grinding feed particles into smaller sizes to enhance nutrient availability and enzymatic digestion in the small intestine. Changes in gizzard size are largely metabolically driven and adaptive, responding to dietary composition (Svihus, 2011). Increased gizzard size is typically associated with higher fiber diets, requiring greater mechanical breakdown of feed (Jha and Mishra, 2021). The higher gizzard yield observed in sorghum- and barley-based diets, regardless of the fat source, may be attributed to the hypertrophic response triggered by the effort required to reduce the particle size of sorghum and the higher insoluble fibre content in barley-based diets. The diameter of sorghum grain typically ranges from 3.1 to 3.6 mm (Qiu et al., 2022). Selle et al. (2019) reported that gizzard weights were higher when sorghum was milled with a larger screen size hammer compared to finer grinding. The geometric characteristics of sorghum may lead to a higher proportion of whole sorghum grains remaining in the final feed mix after grinding, compared to wheat and barley. Whole grain feeding strategies have been shown to effectively promote gizzard development due to their stimulating effect on muscular movement (Silva et al., 2015).

Furthermore, both the type and concentration of fiber are crucial factors in stimulating gizzard muscular development, which leads to improvements in its weight, size, and volume (Bebin et al., 2017; Jha and Mishra, 2021; Zhang et al., 2023). The accumulation of insoluble fiber can slow the passage rate of the fiber fraction (Hetland et al., 2004), which in current study, is partially supported by the numerically lower feed intake observed in birds fed barley-based diets during each growing phase. The sticky nature of barley husk, which is rich in insoluble fiber, can result in the husks easily ending up in diets containing barley (Olkku et al., 2005). As a result, the higher concentration of insoluble fiber in barley, compared to wheat, may partially explain the increased gizzard yield observed in birds fed barley-based diets (Nyman et al., 1984).

Few studies have examined the impact of fat sources on gizzard development. In the current study, broilers fed canola oil exhibited a heavier relative gizzard weight compared to those fed poultry fat and beef tallow. This may be due to the differing fatty acid profiles of the supplemental fat sources. Gaad et al. (2016) observed a linear response in gizzard weight with increasing levels of linoleic acid supplementation, suggesting a potential role of fatty acids in gizzard development. A similar effect of fat source on gizzard weight was noted by Baighi and Nobakht (2017), where increasing canola oil inclusion in the feed increased gizzard relative weight. Canola oil contains a higher amount of unsaturated fatty acids, including monounsaturated, polyunsaturated, and omega-3 fatty acids (91.6 g/100 g oil), compared to beef tallow and poultry fat, which contain 51.6 and 66.9 g/100 g fat, respectively (Pena-Saldarriaga et al., 2020; Ravindran et al., 2016). In contrast, Poorghasemi, et al. (2013) reported no effect of fat source on relative gizzard weight; however, their measurements were based on the full gizzard weight, whereas the current study measured empty gizzard relative weight.

In the current study, feed intake from day 0 to 35 post-hatch was significantly and linearly correlated with the incidence of foot pad dermatitis (*P* = 0.025, r^2^ = 0.70). The lowest feed intake in wheat-beef tallow diets corresponded to the lowest incidence of foot pad dermatitis. A similar correlation between feed intake and foot pad lesion scores was also observed by De Jong et al. (2015). Factors influencing foot pad dermatitis are well documented (Mayne, 2005), with litter quality being the most significant factor. Litter quality, which can be influenced by feed intake and consequently manure excretion, directly affects foot pad health, as it is a form of contact-related skin condition (Hwangbo et al., 2009; Kaukonen et al., 2016).

Previous studies have shown that fat digestibility is influenced by the source of fat, primarily due to its saturation level (Geng et al., 2022; Tancharoenrat et al., 2013). For instance, crude fat digestibility in broiler chickens improved with poultry fat and soybean oil compared to beef tallow from 7 to 21 days post-hatch (Ravindran and Abdollahi, 2021). However, in the present study, fat source had no significant main effect on fat digestibility, nor did it interact with grain source to influence fat digestibility. Similarly, other studies have reported comparable fat digestibility in broilers fed different grain and fat sources (Ahmad et al., 2024; Khatun et al., 2017). Factors that can impact fat digestibility in broilers include age, degree of fat saturation, and fat inclusion level, as documented by Ravindran et al. (2016). Fat digestion ability in broilers increases rapidly during the first week and continues to improve until the third week (Tancharoenrat et al., 2013). The lower secretion of bile acids and lipase in younger birds contributes to poorer fat digestibility, particularly with saturated fatty acid sources (Ketels and De Groote, 1989; Tancharoenrat and Ravindran, 2014). The secretion volume of bile acids and lipase increases weekly. Therefore, the older age of the birds (33 days) and the fat inclusion levels in the current study may explain the absence of dietary factors influencing fat digestibility results.

In the present study, diets containing wheat and beef tallow exhibited high crude protein digestibility, but this trend was also observed in wheat with poultry fat and barley with canola oil. Crude protein digestibility was significantly correlated with the diet pellet durability index (PDI; *P* < 0.01, starter *r* = 0.32; grower *r* = 0.33; finisher *r* = 0.31; and withdrawal *r* = 0.39). Hou (2023) reported that diets containing animal tallow had a higher PDI than those containing plant oils at both high and low oil inclusion levels. In the present study, consistently and regardless of grain source, diets with poultry fat (83.5 %) and beef tallow (82.0 %) had higher PDIs compared to diets with canola oil (80.8 %). Naderinejad et al. (2016) observed a lower digesta passage rate in response to coarser pellets with higher PDIs. Therefore, the improved crude protein digestibility in wheat-based diets with poultry fat and beef tallow may be partially attributed to the slower passage rate in wheat, a ‘viscous grain’, and the high levels of saturated fatty acids in beef tallow. Animal tallow, including beef and poultry fat, is higher in long-chain saturated fatty acids compared to plant oils (Ravindran et al., 2016). Kim et al. (2013) reported a 25-minute increase in intestinal transit time in diets supplemented with beef tallow compared to basal diets. Long-chain saturated fatty acids may prolong transit time due to their lower digestibility and absorption (Netto Cândido et al., 2021). Additionally, the soluble NSP in viscous grains increases digesta viscosity and retention time (Selle et al., 2016). As a result, the prolonged transit time enhances nutrient digestibility in viscous grains more noticeably than in non-viscous grains.

Broilers fed wheat- and barley-based diets exhibited higher starch digestibility compared to those fed sorghum-based diets. These findings are consistent with several studies on starch digestibility in broiler chickens fed sorghum-based diets (Giuberti et al., 2012; Selle et al., 2016; Truong et al., 2016). The challenges of using sorghum in broiler diets have been addressed by Liu et al. (2015a) and Selle et al. (2021). The primary protein component in sorghum, kafirin, may hinder starch digestibility by preventing the swelling of starch granules and obstructing amylase access to starch substrates. This occurs through the formation of linkages between the cysteine-rich periphery of kafirin and the starch. The negative impact of kafirin on starch utilization was demonstrated by Selle et al. (2016), who showed that supplementation with a reducing agent (sodium metabisulphite) can improve diet ME by more than 40 kcal/kg. Starch digestibility was also lower in beef tallow-based diets compared to poultry fat-based diets. The formation of starch-lipid complexes may reduce starch digestibility (Shen et al., 2023). The reduced starch digestibility in beef tallow diets may be attributed to the formation and stabilization of starch-lipid complexes due to the long-chain saturated fatty acids present in beef tallow, compared to canola oil and poultry fat (Wang et al., 2020).

## Conclusion

The results of the current study suggest that, regardless of the supplemental fat source, broiler chickens fed barley- or sorghum-based diets perform comparably to those fed wheat-based diets. However, birds fed sorghum-based diets exhibited compromised starch digestibility, which may partially explain the lower feed efficiency observed in these birds. Additionally, the lower ME content of barley necessitated a higher inclusion of supplemental fat, leading to increased dietary NE levels, which could potentially increase fat-pad deposition.

## Author contributions

**Mengzhu Wang**: Investigation, Data curation, Formal analysis, Writing - original draft. **Shemil Macelline**: Methodology, Data curation, Writing - review & editing. **Peter Selle:** Formal analysis, Review & editing. **Mehdi Toghyani** and **Sonia Liu**: Conceptualization, Methodology, Feed formulation, Funding acquisition, Project administration, Supervision, Writing - review & editing.

## Declaration of competing interest

We declare that we have no financial and personal relationships with other people or organizations that can inappropriately influence our work, and there is no professional or other personal interest of any nature or kind in any product, service and/or company that could be construed as influencing the content of this paper.
